# Botanical inhibitors of SARS-CoV-2 viral entry: a phylogenetic perspective

**DOI:** 10.1038/s41598-023-28303-x

**Published:** 2023-01-23

**Authors:** Caitlin J. Risener, Sunmin Woo, Tharanga Samarakoon, Marco Caputo, Emily Edwards, Kier Klepzig, Wendy Applequist, Keivan Zandi, Shu Ling Goh, Jessica A. Downs-Bowen, Raymond F. Schinazi, Cassandra L. Quave

**Affiliations:** 1 Molecular and Systems Pharmacology, Laney Graduate School, Emory University, Atlanta, GA USA; 2 Center for the Study of Human Health, Emory University, Atlanta, GA USA; 3 The Jones Center at Ichauway, Newton, GA USA; 4 Missouri Botanical Garden, St. Louis, MO USA; 5 Laboratory of Biochemical Pharmacology, Department of Pediatrics and Children’s Healthcare of Atlanta, Emory University, Atlanta, GA USA; 6 Department of Dermatology, Emory University School of Medicine, Atlanta, GA USA

**Keywords:** Antiviral agents, Secondary metabolism

## Abstract

Throughout the SARS-CoV-2 pandemic, the use of botanical dietary supplements in the United States has increased, yet their safety and efficacy against COVID-19 remains underexplored. The Quave Natural Product Library is a phylogenetically diverse collection of botanical and fungal natural product extracts including popular supplement ingredients. Evaluation of 1867 extracts and 18 compounds for virus spike protein binding to host cell ACE2 receptors in a SARS-CoV-2 pseudotyped virus system identified 310 extracts derived from 188 species across 76 families (3 fungi, 73 plants) that exhibited ≥ 50% viral entry inhibition activity at 20 µg/mL. Extracts exhibiting mammalian cytotoxicity > 15% and those containing cardiotoxic cardiac glycosides were eliminated. Three extracts were selected for further testing against four pseudotyped variants and infectious SARS-CoV-2 and were then further chemically characterized, revealing the potent (EC_50_ < 5 µg/mL) antiviral activity of *Solidago altissima* L. (Asteraceae) flowers and *Pteridium aquilinum* (L.) Kuhn (Dennstaedtiaceae) rhizomes.

## Introduction

As of January 2023, there have been over 668 million cases of the coronavirus disease COVID-19, caused by the Severe Acute Respiratory Syndrome Coronavirus 2 (SARS-CoV-2), across the globe, causing over 1.1 million deaths in the USA and 6.7 million worldwide^1^. During the pandemic, many individuals have turned to herbal supplements to prevent COVID-19^2^. There are published in silico studies and a few in vitro studies on these extracts^3–15^, but the science to support the use of these botanicals to prevent viral infection remains incomplete^9,10,13,16^. Our group has conducted field studies in global terrestrial biodiversity hotspots and has amassed a large, targeted collection of plant and fungal species used in traditional medicine for general health (as medicinal foods) and for the treatment of infectious and inflammatory disease^17–19^. Viral entry—in which SARS-CoV-2 attaches to the angiotensin-converting enzyme 2 (ACE2) cell surface receptor found on endothelial cells, pneumocytes (type 1 and 2), and ciliated bronchial epithelial cells—presents an attractive option for preventatives^20^. This is the first extensive investigation of botanical ingredients used in traditional food and medicine systems for their efficacy as viral entry inhibitors for SARS-CoV-2 since the virus emerged in Wuhan, Hubei Province, China, in late 2019^21^.

SARS-CoV-2 is an enveloped RNA virus with a viral spike protein that binds to ACE2 on host cells. Once bound to ACE2, SARS-CoV-2 may enter into the cell through fusion or endocytosis^20^. Additional host cell membrane proteins may alter the spike protein in order to bind to ACE2 (such as TMPRSS2)^20^. For endocytosis, chaperone proteins such as clathrin are also recruited to the membrane to encourage membrane formation and entry into the cell^22^.

There is an urgent need for readily accessible immediate dissemination of orally available therapeutic agents to target viral entry and replication. Since its emergence, various edible and medicinal plants have been proposed as a therapeutic strategy for protecting against infection and treating symptoms^9–13^. There is emerging data that over-the-counter nutritional and dietary supplements may harbor anti-COVID-19 properties^3–7,14,15^, but supporting confirmatory data is still largely lacking. Some natural products (NPs) from traditional Chinese medicine (TCM) preparations are reported to inhibit viral entry and modulate host immune responses^23^. Americans do not typically utilize TCM; however, annually 18–30% of Americans report using botanical dietary supplements^24,25^, with retail sales over $8 billion in 2017^26^. As Americans are looking to prevent infection, many are hoping dietary herbal interventions can be beneficial^13^. Therefore, probing botanical extract libraries can provide further guidance to members of the public seeking to prevent COVID-19 infection through supplements and potentially lessen the severity of COVID-19 infections.

Our group has conducted field studies in the USA, Mediterranean, Africa, Asia, and the Balkans for more than a decade and has amassed a large collection of plant specimens and their extracts which were used medicinally by the local people for general health (such as for medicinal foods) and for the treatment of infectious and inflammatory disease^18,19,27–31^. All 728 species currently in the collection are uniquely linked to ethnobotanical data on their preparation and use in traditional medicine (TM). In total, 2500+ extracts (2497 plant extracts, 83 macrofungal extracts, two algal extracts) representing different tissues from these species, are included in the Quave Natural Products Library (QNPL). Many of the extracts included in the QNPL are created from wild harvested specimens, following WHO guidelines for appropriate plant collection and with necessary permits and permissions^32,33^. The QNPL also has a collection of botanical materials, representing the 40 top selling botanical dietary supplement ingredients^26^ which were acquired from commercial sources, vouchered, botanically authenticated and then extracted. This study utilizes the QNPL as a tool to identify potential natural products with viral entry inhibition effects against SARS-CoV-2 (Supplementary Material 1).

### Screening the QNPL for promising SARS-CoV-2 inhibitors

**Figure 1:**
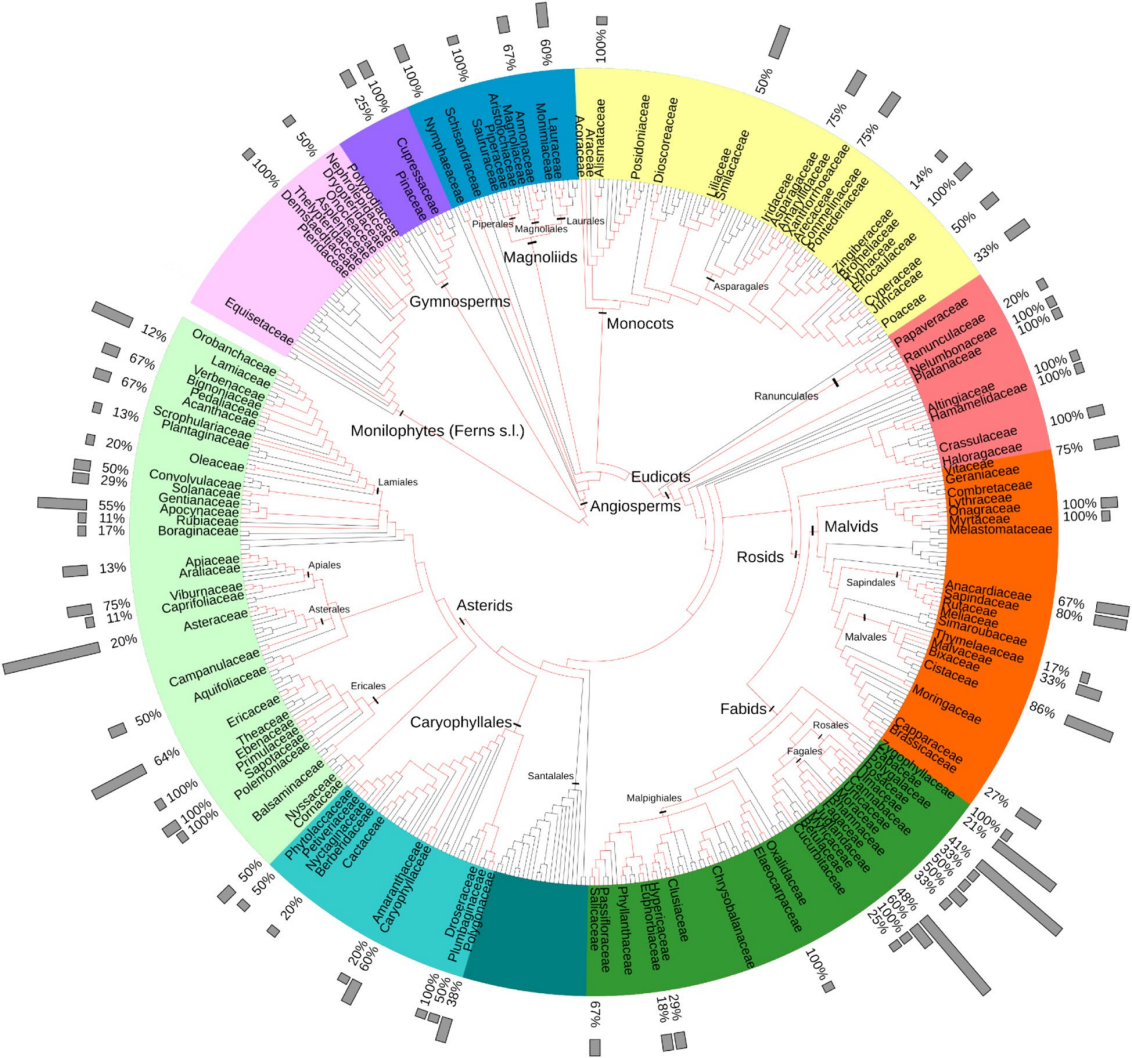
Phylogenetic distribution of plant species tested for SARS-CoV-2 viral entry inhibition. A total of 1867 extracts derived from 660 species (1 Chromista, 27 Fungi, 632 Plantae) across 149 families in the Quave Natural Products Library of plant extracts were screened at a concentration of 20 µg/mL for viral entry inhibition using a wild-type spike (SARS-CoV-2, GeneBank #QHD43416.1) pseudotyped lentivirus model with human embryonic kidney cells expressing human angiotensin converting enzyme 2 (HEK-293T-hACE2). The phylogenetic distribution of plant species demonstrating viral entry inhibition (at ≥ 50% at the screening concentration) were mapped on the family-level maximally resolved complete euphyllophyte phylogenetic tree according to Angiosperm Phylogeny Group IV^68^. Major clades are indicated in different colors and family names are only listed for those genera reported in the present study. Gray bars indicate the percentage of species tested that demonstrated ≥ 50% bioactivity in each family. A high-resolution version of this figure is provided as Supplementary File 4.

A total of 1867 extracts total from the QNPL derived from 660 species (1 Chromista, 27 Fungi, 632 Plantae kingdoms) across 149 families were screened for viral entry inhibition and mammalian cytotoxicity, as well as an additional 18 single compounds that are predominant in botanicals. Hydroxychloroquine (HCQ) was used in the screen as a positive control of viral entry inhibition via the spike-ACE2 complex^34^. Of these, 310 extracts derived from 188 species across 76 families (3 fungi, 73 plants) exhibited ≥ 50% inhibition activity in the wild-type spike pseudotyped model (Fig. 1; Supplementary Materials 2–4). Of these bioactive extracts, 125 extracts derived from 93 plant species across 53 families exhibited ≥ 85% inhibition activity and ≤ 15% cytotoxicity in the wild-type model (Supplementary Material 2). Once these 125 extracts were identified, an interesting pattern emerged, indicating many hits were from species that are known to be cardiotoxic due to a rich composition of cardiac glycosides, including members of the genera *Asclepias* and *Nerium* (Apocynaceae family), *Kalanchoe* (Crassulaceae)*,* and *Drimia* (Asparagaceae). For further selection and testing, we reviewed each extract and consulted the literature to eliminate extracts with those cardiac glycosides or similar compounds.

### Phylogenetic assessment

A phylogenetic assessment of the 185 plant species demonstrating bioactivity (≥ 50% inhibition) identified predominant activity in the asterid families and many species in Fagales and Rosales within the fabid clade of plants (Fig. 1, Supplementary Material 5). A considerable proportion of eudicot families included in this study (57 out of 92 families) exhibited bioactivity. Among those, the fabid clade predominates in terms of number of species that showed viral entry inhibition activity. Fabales, Fagales, Rosales and Malpighiales are the major orders in the fabid clade. Fabaceae is the largest family in this clade, with nine individual species from the QNPL demonstrating bioactivity; however, this represents a relatively small percentage compared to the total number of species included in this study (n = 42) and total species diversity of the family. Fagales is a small clade, with about 1175 species and all four families included in the study showed activity. Five out of six Rosales families demonstrated bioactivity. In total, 58 species from Rosaceae and Fagaceae are included in this study, and 50% of these demonstrated bioactivity (Fig. 1, Supplementary Material 4). Only the Lamiales and some Asteraceae families demonstrated activity in the asterid clade. Lamiid is one of the largest clades in the asterids and 10 out of 15 families studied showed significant antiviral activity. Among the lamiid families, Apocynaceae (n = 6) and Lamiaceae (n = 5) species demonstrated bioactivity. Asteraceae is the largest angiosperm plant family; however, only 12 of 64 species tested from Asteraceae showed activity.

Outside of the asterid and fabid clades, there were a few distinct small families that also demonstrated bioactivity. Two out of nine fern families and two gymnosperms that are included in the study demonstrated activity. Only three out of seven magnoliid families exhibited activity. Bioactivity was low across the monocot species assessed (8 out of 21). Poaceae—one of the largest plant groups in the monocots with high species diversity—had only three species out of nine that exhibited inhibition activity. Only 24 species out of 61 tested in the malvid clade showed bioactivity, with 18 species belonging to Malvales and Sapindales orders. Cistaceae is a small family of the Malvales with 270 species, and all six species included in this study demonstrated bioactivity. The majority of Anacardiaceae (four out of six) and Sapindaceae (four out of five) species from the Sapindales included in the study also demonstrated bioactivity.

### Further testing of hit extracts against emerging variants

**Figure 2:**
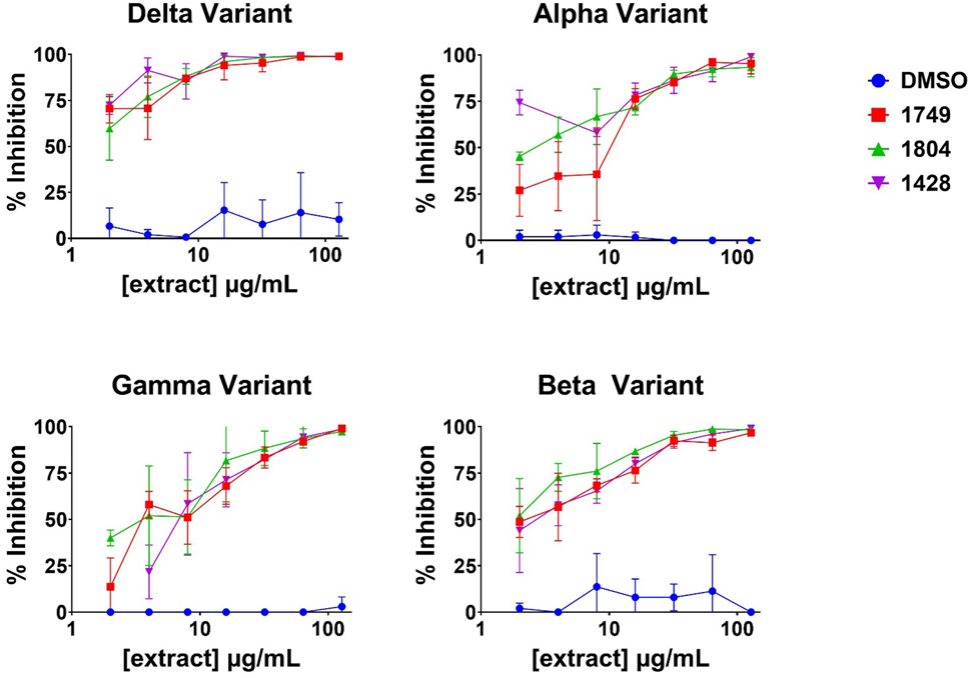
All variants were tested at a titer of 1.2 × 105: Gamma/P.1 variant (BPS Biosciences #78144-1), Beta/B.1.351 variant (BPS Biosciences #78142-1), Delta/B.1.617.2 variant (BPS Biosciences #78215-1), and Alpha/B.1.1.7 variant (BPS Biosciences #78112-1). Concentration-dependent response data (2–128 μg/mL) against SARS-CoV-2 variants for three select extracts (1428, 1749, and 1804) identified as leads for antiviral activity in the SARS-CoV-2 pseudovirion model. Hydroxychloroquine was not included due to study limitations, refer to Tables 1 and 2 for the EC_50_ tested against the wild-type pseudotype virus.

Concentration-dependent response assays against the wild-type spike pseudotyped model were performed at 2 to 64 µg/mL on nine extracts representing seven species of interest. Initial concentration response assays demonstrated that three extracts exhibited potent antiviral activity, low cytotoxicity, a lack of cardiotoxicity concerns based on the literature, and were not identified as common allergens (Table 1, Supplementary Material 6): *Solidago altissima* L. (extract 1428); *Salix nigra* Marshall (extract 1749); and *Pteridium aquilinum* (L.) Kuhn (extract 1804). These extracts were then tested for cell viability, cytotoxicity by lactate dehydrogenase (LDH) assay, and viral entry inhibition at 2 to 128 µg/mL on HEK-293T-hACE2 and HaCaT cells (Table 2). Extracts 1428, 1749, and 1804 demonstrated robust activity at lower concentrations, as well as minimal cytotoxicity at higher concentrations. Concentration-dependent testing of these three extracts in pseudotyped variants (Delta/B.1.617.2, Alpha/B.1.1.7, Gamma/P.1, and Beta/B.1.351) demonstrated bioactivity as well (Table 3, Fig. 2) with EC_50_ values all below 10 µg/mL. In summary, all three lead extracts exhibited activity in pseudotyped models of the wild-type virus and the four variants tested.

**Table 1:** EC_50_ values were calculated by modeled dose–response curves against the wild type SARS-CoV-2 pseudovirion model using non-linear regression.

Extract number	Plant name	Plant part	Extraction method	EC_50_ % Inhibition (µg/mL)	± SEM (µg/mL)	n
DMSO	–	–	–	> 1.25% v/v	–	6
Water	–	–	–	> 1.25% v/v	–	6
Hydroxycholorquine	–	–	–	1.14	0.19	6
220	*Rubus ulmifolius* Schott, Rosaceae	Roots	EtOH 2 × 72 h maceration	22.13	4.47	3
921	*Vaccinium tenellum* Aiton, Ericaceae	Woody stem	MeOH 2 × 72 h maceration	16.61	0.61	3
1104	*Vaccinium myrsinites* Lam., Ericaceae	Roots	MeOH 2 × 72 h maceration	5.22	0.24	3
1428	*Solidago altissima* L., Asteraceae	Flowers	dH_2_O 20 min decoction	2.60	0.23	6
1749	*Salix nigra *Marshall, Salicaceae	Bark	80% EtOH (aq) 2 × 72 h maceration	7.98	1.13	6
1783	*Salix nigra* Marshall, Salicaceae	Roots	80% EtOH (aq) 2 × 72 h maceration	4.85	0.876	3
1804	*Pteridium aquilinum* (L.) Kuhn, Dennstaedtiaceae	Rhizomes	80% EtOH (aq) 2 × 72 h maceration	7.43	1.50	6
1886	*Amorpha fruticosa* L., Fabacaeae	Fruit	80% EtOH (aq) 2 × 72 h maceration	4.59	1.17	3

This experiment was preliminary to help narrow in on potential leads for further characterization.

*SEM* standard error mean.

**Table 2:** EC_50_ values were calculated by modeled dose–response curves against the wild-type SARS-CoV-2 pseudovirion model using non-linear regression.

Extract number	Plant name	Plant part	Extraction method	EC_50_ % Inhibition (µg/mL) ± SEM	EC_50_ % Cytotoxicity (µg/mL) ± SEM		EC_50_ % Cell viability (µg/mL) ± SEM		Selectivity index	
				HEK-293-ACE2	HEK-293-ACE2	HaCaT	HEK-293-ACE2	HaCaT	HEK-293-ACE2	HaCaT
1428	*Solidago altissima* L.	Flowers	dH_2_O 20 min decoction	2.60 ± 0.23	> 1000	> 1000	191.48 ± 13.57	> 1000	73.58	> 384.61
1749	*Salix nigra *Marshall	Bark	80% EtOH (aq) 2 × 72 h maceration	7.98 ± 1.32	> 1000	> 1000	114.08 ± 6.75	241.79 ± 62.50	14.30	30.32
1804	*Pteridium aquilinum* (L.) Kuhn	Rhizomes	80% EtOH (aq) 2 × 72 h maceration	7.43 ± 1.50	> 1000	> 1000	134.04 ± 8.93	145.19 ± 28.93	18.04	19.54
DMSO	–	–	–	> 1000	> 1000	> 1000	> 1000	> 1000	–	–
Water	–	–	–	> 1000	> 1000	> 1000	> 1000	> 1000	–	–
HCQ	–	–	–	1.14 ± 0.19	> 1000	136.54 ± 74.38	109.90 ± 3.73	125.99 ± 7.44	96.00	110.52

These experiments were performed in duplicate with 3 technical replicates for an n of 6.

*SEM* standard error mean, *HCQ* hydroxychloroquine.

Selectivity index calculation: (EC_50_% Cell viability (µg/mL)) divided by (EC_50_% Inhibition (µg/mL)). Accompanying plots can be found in Supplementary Material 6.

**Table 3:** EC_50_ values were calculated by modeled dose–response curves against the wild type SARS-CoV-2 pseudovirion model using non-linear regression.

Pseudotyped virus entry inhibition	EC_50_ (µg/mL)	± SEM	EC_50_ (µg/mL)	± SEM	EC_50_ (µg/mL)	± SEM	EC_50_ (µg/mL)	± SEM
	Extract 1428		Extract 1749		Extract 1804		DMSO	
Wild type	2.60	0.23	7.98	1.32	7.43	1.50	> 128	–
Delta/B.1.617.2	0.75	0.25	0.931	0.36	1.46	0.25	> 128	–
Alpha/B.1.1.7	0.45	0.44	7.67	1.22	2.79	1.22	> 128	–
Gamma/P.1	7.94	1.14	6.54	0.98	4.10	0.62	> 128	–
Beta/B.1.351	2.98	0.45	2.51	0.43	1.73	0.97	> 128	–

This experiment was preliminary to help narrow in on potential leads for further characterization. This experiment was performed once in triplicate except for wild-type (n = 6).

*SEM* standard error mean. Accompanying plots can be found in Fig. 2.

### Antiviral activity shown in infectious virus

**Figure 3:**
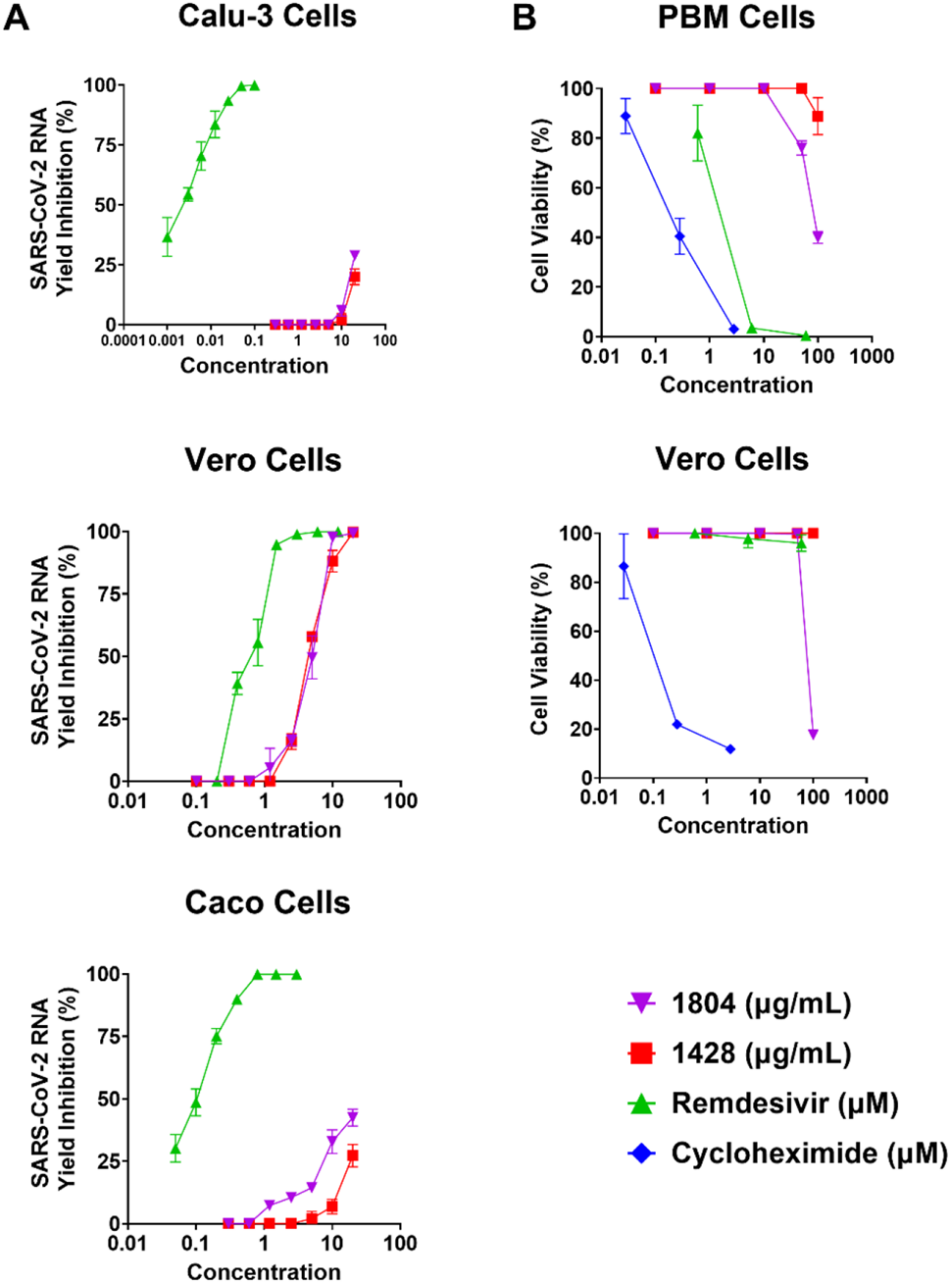
Infectious virus results in different cell lines. (**A**) The antiviral activity of 1804 and 1428 has been further confirmed by virus yield reduction assay using specific qRT-PCR for SARS-CoV-2 to measure the RNA copy number of the virus either 2 days post-treatment (for Vero cells) or 3 days post-treatment (for Calu-3 and Caco-2 cells) in treated-infected cells in a dose response manner. Remdesivir was used as a positive control for both assays. (**B**) Cytotoxicity assays measuring cell proliferation in PBM and Vero cells were performed in parallel to the antiviral assays. Cycloheximide, a known protein synthesis inhibitor, was used as the positive control.

To determine the potential antiviral effects of the top three extracts against in vitro replication of SARS-CoV-2 in cell culture, a confluent monolayer of African Green Monkey kidney (Vero) cells in a 96-well cell culture microplate was treated with 20 μg/mL of compound followed by inoculation with 0.1 multiplicity of infection (MOI) of the virus. This concentration was selected to be consistent with the pseudotyped virus screening against HEK-293T-hACE2 cells. Cells were treated with 12 μg/mL remdesivir as a positive control. Extract 1428 and extract 1804 demonstrated > 99% inhibition at 20 μg/mL in Vero cells, while extract 1749 only demonstrated 45.9% inhibition. The antiviral activity of extract 1428 and extract 1804 was further confirmed by virus yield reduction assay using specific qRT-PCR for SARS-CoV-2 by measuring the RNA copy number of the virus after 2-days post-treatment (for Vero cells) and after 3-days post-treatment (for Calu-3 and Caco-2 cells) in supernatant of treated-infected cells in a concentration–response manner (Table 4, Fig. 3). Extracts 1428 and 1804 showed CC_50_ values of > 100 and > 80 μg/mL respectively, in MTS assays against human peripheral blood mononuclear (PBM) and Vero cells.

**Table 4:** The antiviral activity of 1804 and 1428 has been further confirmed by virus yield reduction assay using specific qRT-PCR for SARS-CoV-2 to measure the RNA copy number of the virus either 2 days post-treatment (for Vero cells) or 3 days post-treatment (for Calu-3 and Caco-2 cells) in treated-infected cells in a dose response manner.

Extract (or compound)	Anti-SARS-CoV-2 activity (Vero)		Anti-SARS-CoV-2 activity (Calu3)		Anti-SARS-CoV-2 activity (Caco2)		Cytotoxicity (CC50)	
	EC_50_	EC_90_	EC_50_	EC_90_	EC_50_	EC_90_	(PBM)	(Vero)
1804 (µg/mL)	4.7	9.5	> 20 (28.9)	> 20	> 20 (42.5)	> 20	80.8	88.1
1428 (µg/mL)	4.5	9.9	> 20 (20.0)	> 20	> 20 (27.2)	> 20	> 100	> 100
Remdesivir (µM)	0.6	1.6	0.002	0.02	0.1	0.4	1.2	> 60.3

Numbers in parentheses indicate percent inhibition at the highest concentration tested. Cytotoxicity assays measuring the cell proliferation in PBM and Vero cells were performed in parallel to the antiviral assays. Accompanying plots can be found in Fig. 3.

### Chemical analysis of top hits

**Figure 4:**
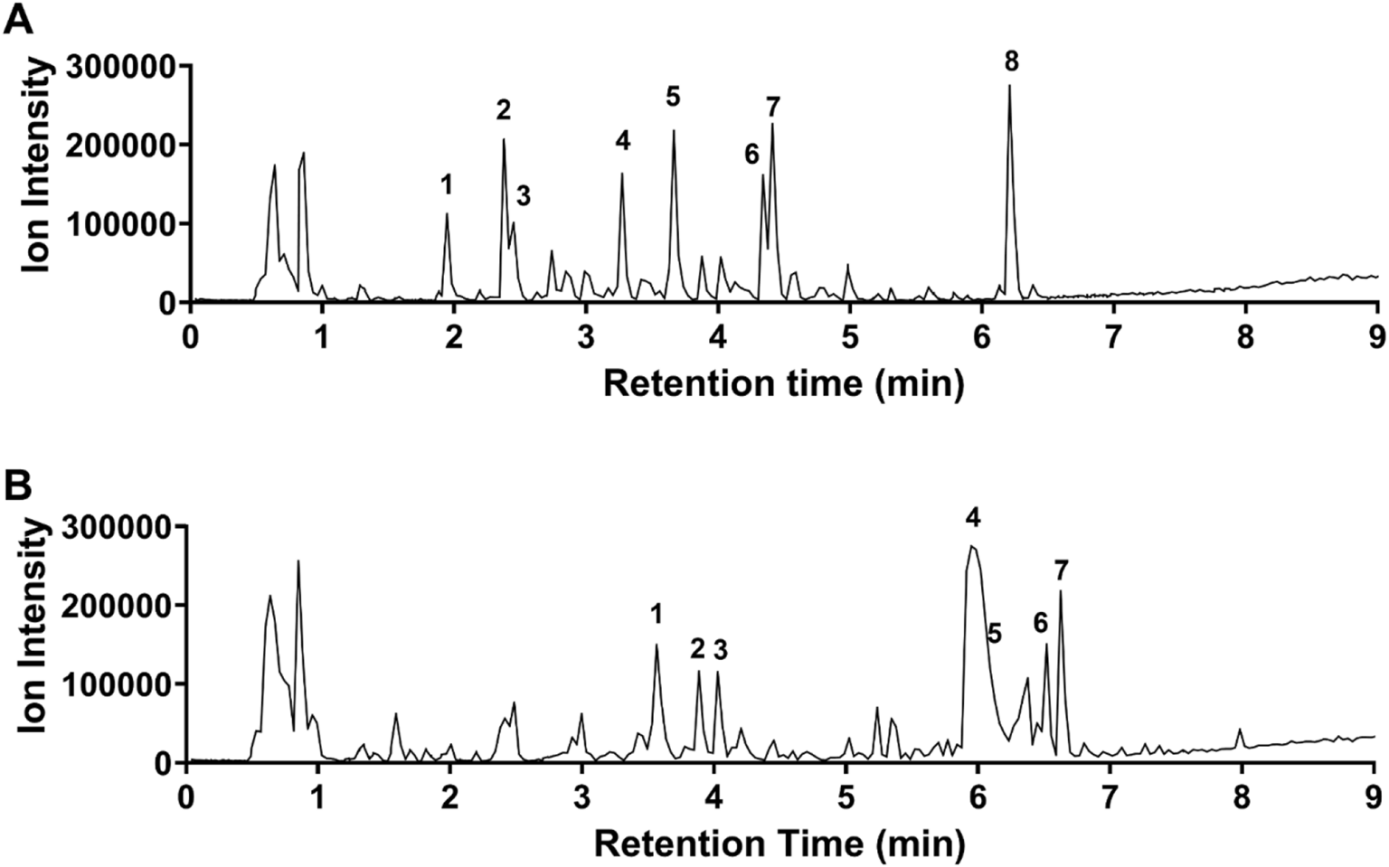
Representative LC–MS base peak ion (BPI) chromatograms of the (**A**) 1804 and (**B**) 1428 extracts. Chromatographic peaks were tentatively annotated with peak numbers corresponding to putative matches annotated in Supplementary Material 7. Supplementary Materials 8 and 9 visualize the chemical compositions and their similarities between the two extracts.

Chemical analysis of these top two candidates was performed using high-resolution mass spectrometry. The major metabolites from both extracts were tentatively identified based on the MS/MS fragmentation data compared with literature, in silico prediction, and web-based databases (Fig. 4, Supplementary Materials 7–9). In extract 1804 (from *Pteridium aquilinum*), a variety of metabolites were detected, including phenylpropanoids, proanthocyanidins, flavonoids, and triterpenes. In extract 1428 (from *Solidago altissima*), phenylpropanoids, glycosidic triterpenoids, and fatty acids were detected as major chemical classes.

## Discussion

Despite increased availability of vaccine doses against SARS-CoV-2 throughout 2021, as of November 14th 2022, only 68% of the United States population is fully vaccinated, while international vaccination rates are reported at 68.2% receiving one dose^1,35,36^. Many countries have access barriers to vaccinating their citizens, and there are many patients that cannot receive the vaccine due to medical and religious reasons. These statistics indicate there is still an urgent need for readily accessible therapeutics and preventatives. Even for those who are vaccinated, there is still a risk of breakthrough cases and the emergence of a variant that may evade the vaccine-mediated immune response, such as the omicron variant^37^. Current SARS-CoV-2 antiviral therapeutic options such as molnupiravir may present challenges to the treatment of pregnant individuals due to toxicity^38^. Apart from remdesivir, these options are still awaiting final approval from the FDA for general use and are currently only approved for emergency use; they are also primarily indicated for patients with early COVID-19. With these considerations, natural product libraries are an essential tool for identifying urgent therapies.

The examination of worldwide biodiversity has historically been a critical part of the drug discovery and has led to the development of many common medications for such applications as pain management, cancer, heart disease, and infection^39^. The use of natural supplements in the United States has increased during the SARS-CoV-2 pandemic^2,40^. The efficacy of these natural products to prevent SARS-CoV-2 infection and the safety of their use remains largely unexplored^13,16^; and the aim of this study was to undertake the largest screen of medicinal plants to identify potential sources of novel therapeutics. This was achieved through use of a medium-throughput robotics platform to test a large, one-of-a-kind botanical extract collection against models of SARS-CoV-2 and for safety in human cells (Supplementary Material 1). This is the first extensive investigation of botanical ingredients used in traditional food and medicine systems for their efficacy as viral entry inhibitors for SARS-CoV-2. Previous studies on natural products and their role against SARS-CoV-2 have been performed in smaller screens (less than 200 plants) or have been primarily screened in silico^3–8^.

An interesting pattern emerged in the screen, revealing many antiviral extracts belong to genera that are rich in cardiotoxic phytochemicals, such as cardenolides and bufadenolides^41^. Recent research corroborates this finding, as it is currently proposed that the Na^+^/K^+^-ATPase is the target of cardiac glycosides in its treatment of SARS-CoV-2. Though these plant extracts may have potential to inhibit SARS-CoV-2 infections, we removed them from our future studies as they are generally not safe for human consumption and their active compound may be toxic.

Both top hits from this study, tall goldenrod (*Solidago altissima*), and eagle fern (*Pteridium aquilinum*), are found in Native American ethnobotany. *S. altissima* flowers were reported in the early twentieth century to be used as burn dressings and applied to ulcers by the Chippewa (Ojibwe) tribes^42^; closely related *Solidago* species are more widely reported as consumed orally in a mixture or tea to treat a variety of diseases^43^. It is important to note as a member of the Asteraceae family, there is a risk of allergenicity, but that is more common with ragweed than goldenrod^44^. *P. aquilinum* is consumed in the diet across many Native American tribes, and it has a long history of varied use such as materials for basket weaving, cooking tools, tonics, antiseptic, and anthelminthic^45,46^. Additionally, *P. aquilinum* has global ethnobotanical uses as a food^47^. While it is uncommon within the literature to have an allergic response to *P. aquilinum*, preparation is important to reduce toxicity. *P. aquilinum* rhizomes and shoots are traditionally cooked before consumption, whereas mature fronds are not typically consumed^48–55^.

As a result of the MS data analysis, extract 1804 (*Pteridium aquilinum*) was found to contain proanthocyanidins including dimeric procyanidins, trimeric procyanidin, and prodelphinidin. In silico molecular docking studies of a proanthocyanidin against 3C-like protease was reported previously^56^, but additional antiviral evaluation using isolated compounds is still needed. Moving forward, there is vast chemical diversity within extract 1428 (*Solidago altissima*) awaiting exploration. Additionally, future studies should explore the antiviral effect of specific proanthocyanidins found in extract 1804. Interestingly, besides these two extracts, extracts 921 (*Vaccinium tenellum*), 1104 (*Vaccinium myrsinites*), 1783 (*Salix nigra*), and 1886 (*Amorpha fruiticosa*) (Table 1) exhibited potent antiviral activity in the pseudotyped model, and their genera are known to be rich in the proanthocyanidins class of compounds^57–59^. Extract 1804 and 1428 are from two different plant families, leading to distinct chemical diversity. Though proanthocyanidins are a common class among these hits, the bioactive compounds must be isolated before discussing the chemical relationship between the extracts.

Our study is not without limitations. First, screening with complex mixtures from plant extracts can produce variable results based upon the health of the plant when collected, location collected, and the extraction methods. Our group addressed this issue during validation by collecting fresh samples at the same season with similar maturity as previous collections. Additionally, our initial screen in the pseudotyped model was limited to two technical replicates with no biological replicates due to cost constraints of the model. With this large limitation acknowledged, we strengthened the data of lead hits with robust concentration–response testing in both pseudotyped and infectious virus models requiring three technical replicates and two biological replicates. Finally, ACE2 is involved in numerous processes in the body, most importantly regulating the effects of angiotensin 2, potentially causing off-target problems; however, there are studies supporting ACE2 inhibitors decreasing damage to the respiratory system^60,61^. It is also important to emphasize that though our model utilizes Spike-ACE2, this study does not report a determined mechanism of action.

Determining the mechanism of action in which these compounds prevent entry into SARS-CoV-2 is beyond the scope of this study. Identifying the bioactive compounds from each plant is essential for mechanism of action studies and will allow for future in silico modeling. Though our studies utilize a model with Spike and ACE2, we plan to explore additional components in cellular entry, such as TMPRSS2^62^. Additionally, due to the quickly evolving nature of SARS-CoV-2 infections globally, there are new variants of interest that we were not able to examine within our study^63^, but plan to include the most relevant variants in future characterizations of these natural products. We anticipate bioassay guided fractionation will increase potency in additional cell lines (Calu-3 and Caco-2, for example) as the active components’ concentration increases.

This study is the largest screen of natural products derived from traditional medicinal plants utilized to identify potential compounds to inhibit SARS-CoV-2 infection. A screen of this size provides value from a phylogenetic perspective, offering insight into plant families meriting further study. Moving forward, targeted natural product libraries such as the QNPL can be leveraged to identify tool compounds or potential therapeutics in additional models of infectious diseases.

## Methods

### Botanical terminology

All plant names are in accordance with World Flora Online (http://www.worldfloraonline.org). Plant family assignments follow the Angiosperm Phylogeny Group IV guidance^64^.

### Natural products chemical library creation

The Quave Natural Products Library (QNPL) was created from different parts of 725 plant and 23 fungal species through organic and aqueous (distilled H_2_O or dH_2_O) extraction. Following extraction, 1867 extract samples were dried through rotary evaporation and lyophilization and stored at - 20 °C until being dissolved in either DMSO or water for creation of a stock concentration for testing. All source plants and fungi were collected with appropriate permissions on public or private lands and authenticated by a qualified botanist (co-author, T. Samarakoon) and deposited with herbaria, with most stored at the Emory University Herbarium (GEO), where they have been digitized and are accessible for viewing via the SERNEC portal^65^. Retention vouchers were also created of all materials before and after processing by grinding in a Wiley mill. Herbarium voucher specimen deposit numbers are reported with bioactivity data in Supplementary Material 4.

### Cells and culture conditions

Human embryonic kidney cells expressing human angiotensin converting enzyme 2 (HEK-293T-hACE2) cell line were obtained from BEI Resources (#NR-52511). HaCaT (human keratinocyte) cells were provided by Dr. Brian Pollack. Cells were maintained in a humidified, 5% CO_2_, 37 °C incubator in DMEM with 10% Fetal Bovine Serum and 1X Penicillin–Streptomycin. Cells were passaged at 70–80% confluency. For all assays, cells were seeded in the same media at 1 × 10^5^ cells/well into tissue culture-treated 384-well plates and incubated for 24 h. On the day of testing, media was removed and replaced with fresh media containing extracts (concentrations described below).

Vero (African Green Monkey Kidney, ATCC CCL-81), Calu-3 (Human Lung Carcinoma, ATCC HTB-55) and Caco-2 (Human Colorectal Adenocarcinoma, obtained from Dr. Saul Karpen, Paul Dawson and Anu Rao, ATCC HBT-37) cell lines were also used in this study. Vero cells were maintained in a humidified, 5% CO_2_, 37 °C incubator in MEM containing 10% heat inactivated fetal bovine serum (FBS), while Calu-3 and Caco-2 cells were maintained and cultured in EMEM containing 10% FBS. The cells were incubated at 37 °C in the presence 5% CO_2_. At the time of virus inoculation and antiviral assays, the concentration of FBS was reduced to 2%.

### Infectious virus

SARS-CoV-2 (Isolate USA-WA1/2020) was provided by BEI Resources (Manassas, VA). SARS-CoV-2 has been propagated in Vero cell line and titrated by TCID_50_ method followed by storage of aliquots at - 80 °C until further use in the experiments.

### Spike pseudotyped SARS-CoV-2 assay

The viral spike protein recognizes and attaches to the angiotensin-converting enzyme 2 (ACE2) receptor found on the surface of type I and II pneumocytes, endothelial cells, and ciliated bronchial epithelial cells. A spike (SARS-CoV-2, GeneBank #QHD43416.1) pseudotyped lentivirus (Luc Reporter) model (BPS Biosciences, #79942) was used in the original screen of the QNPL natural products library (20 µg/mL in vehicle; 0.2% DMSO or water in final well volume) to identify viral entry inhibitors with human HEK-293T-hACE2 cells. Extracts were added to media and fresh cells, then incubated for 30 min before adding pseudotyped lentivirus. Samples were then incubated for 48 h. At 48 h, media was removed and luciferase data was collected with PerkinElmer BriteLite Plus Reporter Gene Assay System (Ref. #6066761) per manufacturer protocol. Due to the use of phenol red in the media, supernatant was removed and replaced with 10 µL dPBS with calcium and magnesium (VWR; #97063-660) + 10 µL luciferase substrate mixture. Percent inhibition was calculated in comparison to the vehicle control. Positive controls included wells treated with a bald-VSV pseudotyped virus (BPS Biosciences, #79943) and hydroxychloroquine (64 µg/mL in water). Screening tests were performed in duplicate with one technical replicate due to cost restraints of the study. Results from the screening study were used to narrow down hits for analysis of concentration dependent activity (512 or 64–2 µg/mL, < 0.2% DMSO or water in final well volume). All concentration-dependent testing was performed with three technical replicates and repeated thrice (three biological replicates). All concentration-dependent testing was performed with three technical replicates and three biological replicates.

### Cytotoxicity assays

In parallel to the pseudotyped screening, all extracts were tested on HEK-293T-hACE2 (20 µg/mL in vehicle; 0.2% DMSO or water in final well volume) and incubated for 48 h. Cytotoxicity was measured using a lactate dehydrogenase (LDH) assay (G Biosciences, #786–210) per manufacturer protocol. Additional cytotoxicity assays were run in a serial dilution at 512–2 µg/mL or 64–2 µg/mL (< 0.2% DMSO or water in final well volume) in the same LDH assay. Cell viability assays on HEK293T-hACE2 cells and HaCaT cells were performed with the CellTiter-Glo® Luminescent Cell Viability Assay (Promega) according to manufacturer protocol. MTS assay were performed on various cell lines (PBM and Vero) using the CellTiter 96® Non-Radioactive Cell Proliferation (Promega) kit as previously described^66^. Briefly, cell proliferation, with or without test compounds, was measured after four days’ incubation. Cytotoxicity was expressed as the concentration of test compounds that inhibited cell proliferation by 50% (CC50) and calculated using the Chou and Talalay method^66^. For MTS assays, the half maximal effective concentration (EC_50_) was calculated using GraphPad PRISM for Windows, version 5 (GraphPad Software Inc., San Diego, CA, 2005).

### Virus kinetic replication assay

To determine the kinetic replication of SARS-CoV-2 in each cell line, a confluent monolayer of Vero, Calu-3 and Caco-2 cell lines in a 96-well cell culture microplate were inoculated at an MOI of 0.1 and the yield of progeny virus production was assessed in different time points using a specific qRT-PCR assay for SARS-CoV-2 for each cell line. Briefly, a one-step qRT-PCR was conducted in a final volume of 10 μL containing extracted viral RNA, probe/primer mix, and qScript-Tough master mix (Quantibio, USA). Quantitative PCR measurement was performed using LightCycler® 480 PCR system (Roche, Germany) according to manufacturer’s protocol.

### Infectious antiviral activity assays

#### Antiviral evaluation

To determine the potential antiviral effects of the extracts against in vitro replication of SARS-CoV-2 in cell culture, a confluent monolayer of Vero cells in a 96-well cell culture microplate was treated with 20 μg/mL of compound followed by inoculation with 0.1 MOI of the virus. To assess the antiviral activity, a virus yield reduction assay using specific qRT-PCR for each virus was performed. The half maximal effective concentration (EC_50_) was calculated using GraphPad PRISM for Windows, version 9 (GraphPad Software Inc., San Diego, CA, 2005).

#### Concentration dependent antiviral assay

The antiviral activity of extracts 1428 and 1804 have been further confirmed by virus yield reduction assay using specific qRT-PCR for SARS-CoV-2 by measuring the RNA copy number of the virus after 2-days post-treatment (for Vero cells) and after 3-days post-treatment (for Calu-3 and Caco-2 cells) in supernatant of treated-infected cells in a dose response manner (extracts tested at 0.3–20 μg/mL, remdesivir tested at 1–100 μM). One step qRT-PCR was carried out in a final volume of 10 μL containing extracted viral RNA, specific probe/primer mix and qScript-Tough master mix (Quantibio, USA). Quantitative PCR measurement was performed using LightCycler® 480 PCR system (Roche, Germany) according to manufacturer’s protocol. The half maximal effective concentration (EC_50_) was calculated using GraphPad PRISM for Windows, version 9 (GraphPad Software Inc., San Diego, CA, 2005).

### Phylogenetic analysis

The phylogenetic backbone of plants used to map activity against SARS-CoV-2 was obtained from Gastauer & Meira Neto, 2017 as the text file R20160,415_euphyllophyte.new (Newick format). The resulting high-resolution tree only contains branches with confidence levels greater than 80%, determined by bootstrap values or posterior probabilities from Bayesian analysis. Plant families were graphically displayed using iTOL^67^. The tree contains 13 gymnosperm, 37 monilophyte (fern and horsetail) families, and all 64 orders and 416 Angiosperm families recognized by APG IV^64,68^. Percentages of studied genera are calculated based on the number of genera and species for each family according to the angiosperm working group version 14^69^.

### Chemicals

Optima® LC–MS grade acetonitrile and water, containing 0.1% formic acid respectively, and methanol were purchased from Fisher Scientific (Pittsburgh, PA, USA) and Supelco (Bellefonte, PA, USA).

### LC–MS analysis

The dried extracts of 1749, and 1428 were dissolved with MeOH/H_2_O (5:5, v/v) by 10.0 mg/mL concentration for LC–MS analysis. The 1428 extracts (7.8 g) were further subjected to column chromatography on an HP-20 resin (3 × 14 cm id) and eluted by water, 50% methanol, methanol, and acetone to yield 4 fractions, and each fraction was dissolved with MeOH/dH_2_O (5:5, v/v) by 5.0 mg/mL for the LC–MS analysis. The LC–MS analysis was performed on an Agilent 1290 Infinity II UHPLC system coupled to an Agilent 6545XT QTOFMS (Agilent Technologies, Santa Clara, CA, USA), which was equipped with a Dual AJS ESI Ion Source (Agilent Technologies). Chromatographic separations were performed on a Zorbax Eclipse XDB-C18 (100 × 2.1 mm, 1.8 μM) column coupled with Zorbax Eclipse XDB-C18 (5 × 2.1 mm, 1.8 μM) guard column. The mobile phase was comprised of H_2_O (A) and acetonitrile (B), both of which were acidified with 0.1% formic acid. The column temperature and sample organizer were maintained at 40 °C and 15 °C, respectively. A stepwise gradient method at a constant flow rate of 0.4 mL/min was used to elute the column with the following conditions: 5–5% B (0.0–0.5 min); 5–25% B (0.5–4.0 min); 25–60% B (4.0–7.0 min); 60–100% B (7.0–9.0 min); and 100–100% B (9.0–10.5 min), followed by a return to the starting conditions at 10.6 min and 1.4 min of reconditioning the column (total runtime of 12.0 min). Analyses of the samples (2.0 μL, injection volume) were performed in the negative ion mode in both profile and centroid mode. The ESI conditions were set as follows: the capillary voltage was 4.0 kV, the nozzle voltage was 2000 V for negative mode, the fragmentor was 100 V, the drying gas temperature and flow were set to 325 °C and 13 L/min, respectively, and sheath gas temperature and flow were 275 °C and 12 L/min, respectively, and the nebulizer was operating at 35 psi. Nitrogen served both as the nebulizer gas and the dry gas. The Auto-MS/MS mode was used with an MS range of m/z 100–1700 and an MS2 range of m/z 50–1700, at 7 spectra/s and 5 spectra/s, respectively. The narrow isolation (~ 1.3 *m/z*) width was used. The collision energy was set by the formula based on the *m/z* and charge of the precursor (condition 1: slope of 3.8 and an offset of 20, condition 2: slope of 2.0 and an offset of 6). The maximum precursors per cycle are set to 5, with the absolute precursor threshold set to 500 (relative threshold 0.015%) and active exclusion after 3 scans and released after 0.1 min were performed. MassHunter Workstation Acquisition B.10.00 software and MassHunter Qualitative Analysis 10.0 software (Agilent Technologies) were used for acquiring and processing MS data.

### Molecular networking

A molecular network was created using the online workflow (https://ccms-ucsd.github.io/GNPSDocumentation/) on the GNPS website (http://gnps.ucsd.edu)^70^. The precursor ion mass tolerance was set to 0.02 Da and a MS/MS fragment ion tolerance of 0.05 Da, and then a network was created where edges were filtered to have a cosine score above 0.6 and more than 4 matched peaks. Further, edges between two nodes were kept in the network if and only if each of the nodes appeared in each other’s respective top 10 most similar nodes. Finally, the maximum size of a molecular family was set to 100, and the lowest scoring edges were removed from molecular families until the molecular family size was below this threshold. The spectra in the network were then searched against GNPS’ spectral libraries. All matches kept between network spectra and library spectra were required to have a score above 0.7 and at least 4 matched peaks^70^. The generated molecular network was visualized using Cytoscape 3.8.2. Additional information: MSI level 1—annotated by standard compound, MSI level 2—annotated by MS/MS spectral matching to the GNPS spectral library, MSI level 3—annotated by comparison of relative retention time and MS data with previous studies and characterized compound classes^71^.

### Statistical analysis

All statistics unless otherwise noted were performed using Prism 9. All negative % values in analysis were normalized to 0. A nonlinear regression was performed to determine the extract concentration at which pseudo-virus entry was 50% inhibited (IC_50_). The top was constrained to 100 and bottom to 0, therefore reported IC_50_ is considered absolute. D’Agostino-Pearson omnibus normality test was performed to ensure Gaussian distribution of the data points (P < 0.05, not significant if normality test passed). Range for the nonlinear regression was 0.01–1000 µg/mL with 2000 points to define the curve. Significance of data at each treatment concentration for both viral entry inhibition and cytotoxicity was determined using a one-way ANOVA followed by Dunnett’s multiple comparisons test comparing the mean of the % inhibition or % cytotoxicity for each treatment concentration of each extract to the average of the corresponding vehicle control treatment.

## Reporting summary

Further information on research design is available in the Nature Research Reporting Summary linked to this paper.

## Supplementary Information

Supplementary Information 1.

Supplementary Information 2.

Supplementary Information 3.

Supplementary Information 4.

Supplementary Information 5.

Supplementary Information 6.

Supplementary Information 7.

Supplementary Information 8.

Supplementary Information 9.

## Data Availability

The authors declare that the data supporting the findings of this study are available within the paper and its Supplementary Information files.
