# Supplementary material for: Botanical inhibitors of SARS-CoV-2 viral entry: a phylogenetic perspective
Source: Sci Rep. 2023 Jan 23;13:1244. doi: 10.1038/s41598-023-28303-x (PMC9868516; doi:10.1038/s41598-023-28303-x)

# Supplementary Material 1:

Visual schematic of the experimental pipeline in this study. Schematic created with Biorender.com


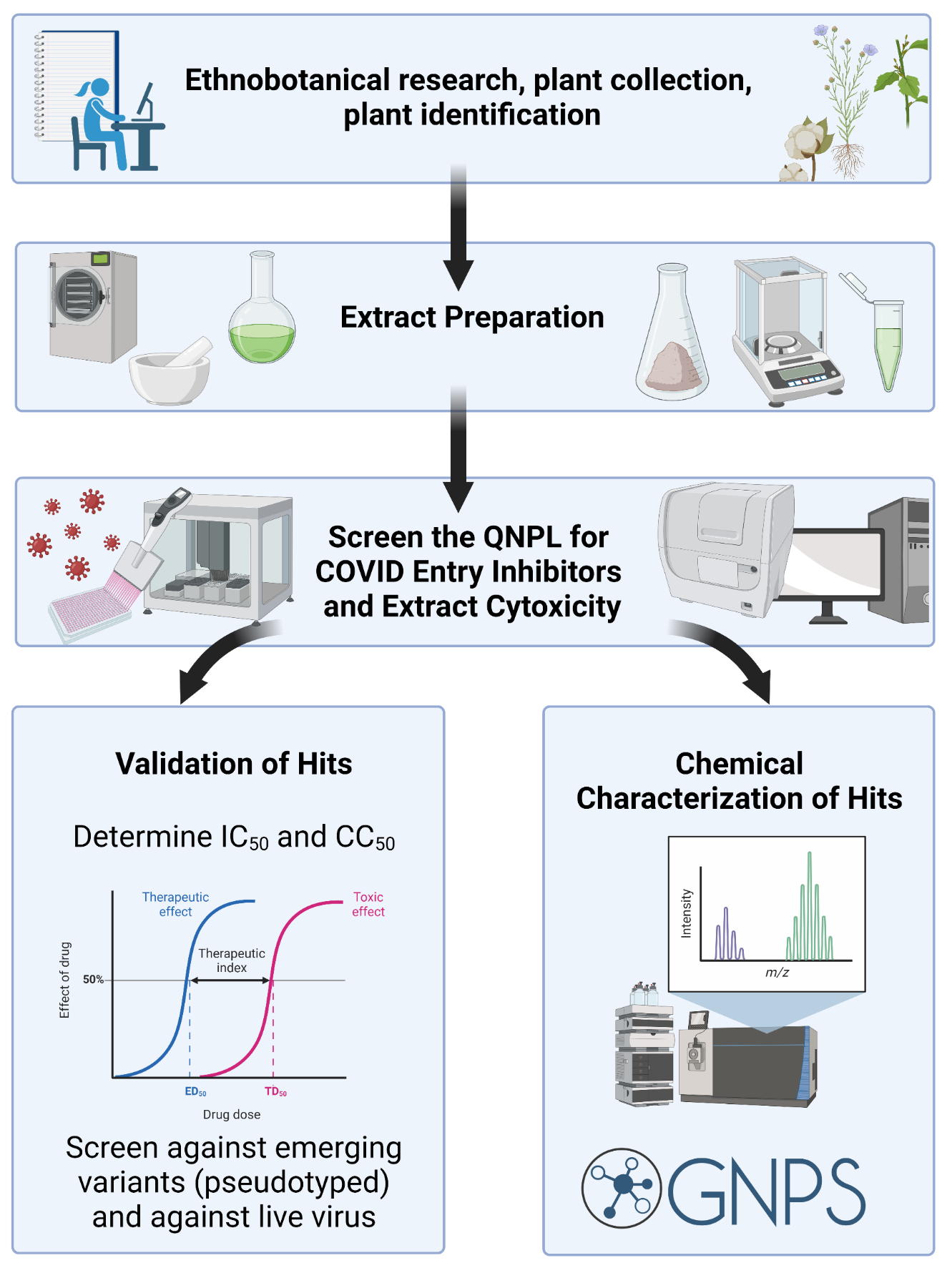

Supplement: Supplementary file 1 — Supplementary Information 1. [file 41598_2023_28303_MOESM1_ESM.docx]
