# Supplementary material for: Botanical inhibitors of SARS-CoV-2 viral entry: a phylogenetic perspective
Source: Sci Rep. 2023 Jan 23;13:1244. doi: 10.1038/s41598-023-28303-x (PMC9868516; doi:10.1038/s41598-023-28303-x)

# Supplementary Material 2:

Screening results (all extracts tested at 20 μg/mL) for activity in the pseudotyped model and counter screening for cytotoxicity. Prioritized extracts (% Inhibition of VLP >85%; cytotoxicity <15%) for further study are highlighted in the red box.


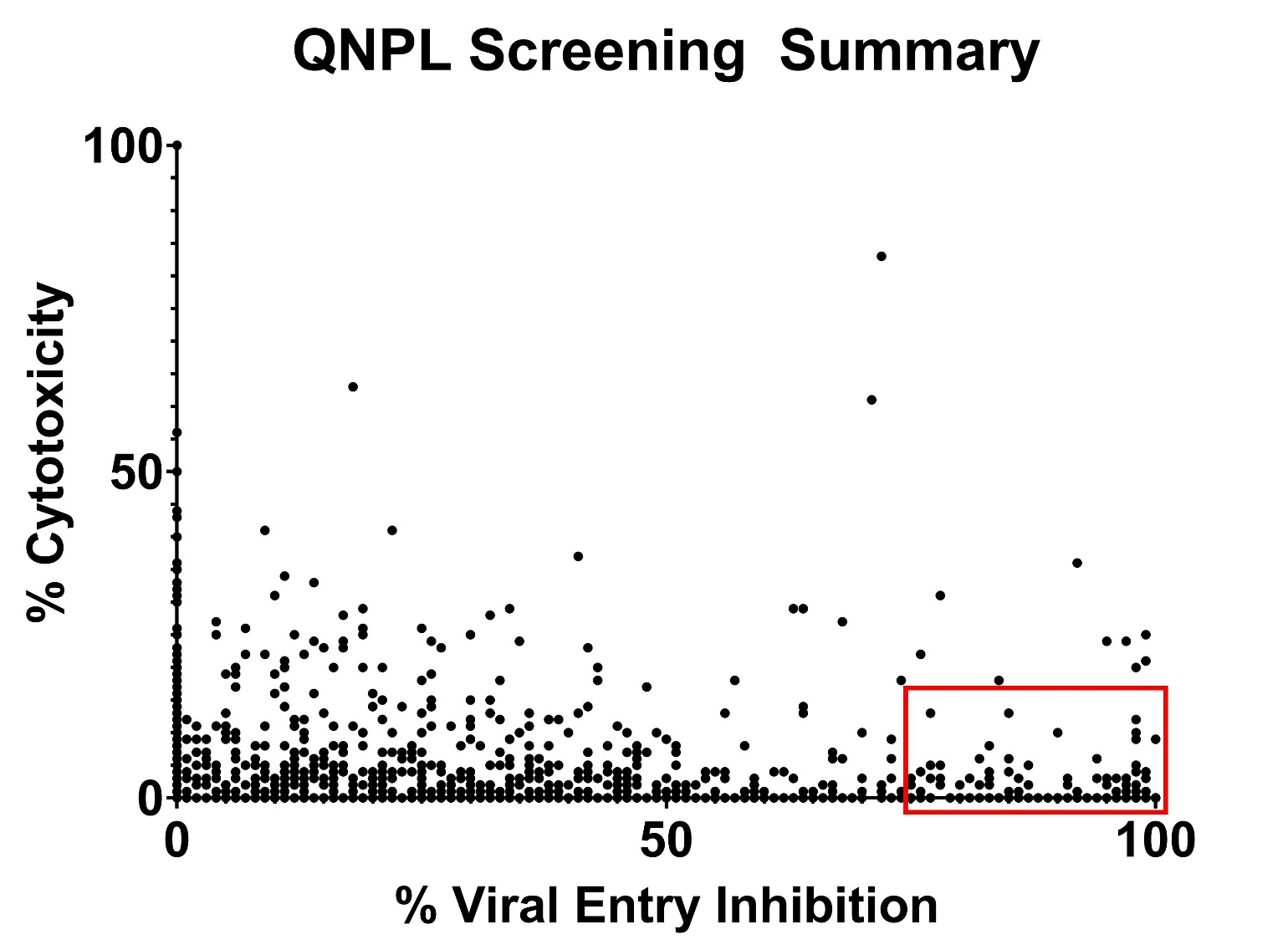

Supplement: Supplementary file 2 — Supplementary Information 2. [file 41598_2023_28303_MOESM2_ESM.docx]
