# Supplementary material for: Botanical inhibitors of SARS-CoV-2 viral entry: a phylogenetic perspective
Source: Sci Rep. 2023 Jan 23;13:1244. doi: 10.1038/s41598-023-28303-x (PMC9868516; doi:10.1038/s41598-023-28303-x)
Supplement: Supplementary file 3 — Supplementary Information 3. [file 41598_2023_28303_MOESM3_ESM.docx]

**Supplementary Material 3:**

Summary of plant species that were hits in the primary screen for antiviral activity in the SARS-CoV-2 pseudovirion (≥50% inhibition). Total number of species in each family sourced from the Angiosperm Phylogeny Website^1^. Note that this table only includes those families included in the antiviral screen. Subtotal counts are not representative of all species present within each order.

| Order | Family | Total N° Species in Family | N° Species in Family Tested | % of Total Species Tested | N° Species with Activity | % of Species Tested with Activity |
| --- | --- | --- | --- | --- | --- | --- |
| Acorales | Acoraceae | 2 | 1 | 50% | 0 | 0% |
| Alismatales | Alismataceae | 115 | 1 | 0.87% | 1 | 100% |
|  | Araceae | 3645 | 3 | 0.08% | 0 | 0% |
|  | Posidoniaceae | 9 | 1 | 11.11% | 0 | 0% |
| *Subtotals* | | *3771* | *5* | *0.13%* | *1* | *20%* |
| Apiales | Apiaceae | 3820 | 21 | 0.55% | 3 | 13% |
|  | Araliaceae | 1450 | 6 | 0.41% | 0 | 0% |
| *Subtotals* | | *5270* | *27* | *0.51%* | *3* | *11%* |
| Aquifoliales | Aquifoliaceae | 500 | 4 | 0.80% | 2 | 50% |
| Arecales | Arecaceae | 2585 | 4 | 0.15% | 3 | 75% |
| Asparagales | Amaryllidaceae | 1605 | 3 | 0.19% | 0 | 0% |
|  | Asparagaceae | 2595 | 7 | 0.27% | 3 | 75% |
|  | Iridaceae | 2120 | 1 | 0.05% | 0 | 0% |
|  | Xanthorrhoeaceae | 900 | 1 | 0.11% | 0 | 0% |
| *Subtotals* | | *7220* | *12* | *0.17%* | *3* | *25%* |
| Asterales | Asteraceae | 25040 | 64 | 0.26% | 12 | 20% |
|  | Campanulaceae | 2380 | 1 | 0.04% | 0 | 0% |
| *Subtotals* | | *27420* | *65* | *0.24%* | *12* | *18%* |
| Austrobaileyales | Schisandraceae | 92 | 1 | 1.09% | 0 | 0% |
| Boraginales | Boraginaceae | 1793 | 6 | 0.33% | 1 | 17% |
| Brassicales | Brassicaceae | 3973 | 12 | 0.30% | 3 | 27% |
|  | Capparaceae | 480 | 1 | 0.21% | 0 | 0% |
|  | Moringaceae | 12 | 1 | 8.33% | 0 | 0% |
| *Subtotals* | | *4465* | *14* | *0.31%* | *3* | *21%* |
| Caryophyllales | Amaranthaceae | 2050 | 5 | 0.24% | 1 | 20% |
|  | Cactaceae | 1866 | 1 | 0.05% | 0 | 0% |
|  | Droseraceae | 205 | 1 | 0.49% | 1 | 100% |
|  | Nyctaginaceae | 405 | 1 | 0.25% | 0 | 0% |
|  | Petiveriaceae | 13 | 1 | 7.69% | 0 | 0% |
|  | Phytolaccaceae | 32 | 2 | 6.25% | 1 | 50% |
|  | Plumbaginaceae | 836 | 2 | 0.24% | 1 | 50% |
|  | Polygonaceae | 1110 | 8 | 0.72% | 3 | 38% |
| *Subtotals* | | *6517* | *21* | *0.32%* | *7* | *33%* |
| Commelinales | Commelinaceae | 652 | 1 | 0.15% | 0 | 0% |
|  | Pontederiaceae | 33 | 2 | 6.06% | 0 | 0% |
| *Subtotals* | | *685* | *3* | *0.44%* | *0* | *0%* |
| Cornales | Cornaceae | 180 | 4 | 2.22% | 2 | 50% |
|  | Nyssaceae | 22 | 1 | 4.55% | 0 | 0% |
| *Subtotals* | | *202* | *5* | *2.48%* | *2* | *40%* |
| Cucurbitales | Cucurbitaceae | 1000 | 5 | 0.5% | 0 | 0% |
| Cupressales | Cupressaceae | 133 | 2 | 1.5% | 2 | 100% |
| Dioscoreales | Dioscoreaceae | 870 | 1 | 0.11% | 0 | 0% |
| Dipsacales | Caprifoliaceae | 890 | 9 | 1.01% | 1 | 11% |
|  | Caryophyllaceae | 2200 | 5 | 0.23% | 3 | 60% |
|  | Viburnaceae | 200 | 4 | 2% | 3 | 75% |
| *Subtotals* | | *3290* | *18* | *0.55%* | *7* | *39%* |
| Equisetales | Equisetaceae | 20 | 1 | 5% | 0 | 0% |
| Ericales | Balsaminaceae | 1001 | 1 | 0.10% | 0 | 0% |
|  | Ebenaceae | 855 | 1 | 0.12% | 1 | 100% |
|  | Ericaceae | 4215 | 11 | 0.26% | 7 | 64% |
|  | Polemoniaceae | 385 | 1 | 0.26% | 1 | 100% |
|  | Primulaceae | 2590 | 2 | 0.08% | 0 | 0% |
|  | Sapotaceae | 1100 | 2 | 0.18% | 2 | 100% |
|  | Theaceae | 195 | 1 | 0.51% | 0 | 0% |
| *Subtotals* | | *10341* | *19* | *0.18%* | *11* | *58%* |
| Fabales | Fabaceae | 19580 | 42 | 0.21% | 9 | 21% |
|  | Polygalaceae | 1236 | 2 | 0.16% | 0 | 0% |
| *Subtotals* | | *20816* | *44* | *0.21%* | *9* | *20%* |
| Fagales | Betulaceae | 145 | 4 | 2.76% | 1 | 25% |
|  | Fagaceae | 730 | 25 | 3.42% | 12 | 48% |
|  | Juglandaceae | 51 | 5 | 9.80% | 3 | 60% |
|  | Myricaceae | 57 | 1 | 1.75% | 1 | 100% |
| *Fagales Subtotals* | | *983* | *35* | *3.56%* | *17* | *49%* |
| Gentianales | Apocynaceae | 4555 | 11 | 0.24% | 6 | 55% |
|  | Gentianaceae | 1750 | 5 | 0.29% | 0 | 0% |
|  | Rubiaceae | 13465 | 9 | 0.07% | 1 | 11% |
| *Subtotals* | | *19770* | *25* | *0.13%* | *7* | *28%* |
| Gerianales | Geraniaceae | 866 | 3 | 0.35% | 0 | 0% |
| Lamiales | Acanthaceae | 4320 | 3 | 0.07% | 2 | 67% |
|  | Bignoniaceae | 790 | 3 | 0.38% | 2 | 67% |
|  | Lamiaceae | 7280 | 42 | 0.58% | 5 | 12% |
|  | Oleaceae | 615 | 5 | 0.81% | 1 | 20% |
|  | Orobanchaceae | 2025 | 2 | 0.10% | 0 | 0% |
|  | Pedaliaceae | 70 | 1 | 1.43% | 0 | 0% |
|  | Plantaginaceae | 1900 | 8 | 0.42% | 1 | 13% |
|  | Scrophulariaceae | 1880 | 4 | 0.215 | 0 | 0% |
|  | Verbenaceae | 918 | 1 | 0.11% | 0 | 0% |
| *Subtotals* | | *19798* | *69* | *0.35%* | *11* | *16%* |
| Laurales | Lauraceae | 2500 | 5 | 0.20% | 3 | 60% |
|  | Monimiaceae | 200 | 1 | 0.50% | 0 | 0% |
| *Subtotals* | | *2700* | *6* | *0.22%* | *3* | *50%* |
| Liliales | Liliaceae | 610 | 1 | 0.16% | 0 | 0% |
|  | Smilacaceae | 210 | 8 | 3.81% | 4 | 50% |
| *Subtotals* | | *820* | *9* | *1.10%* | *4* | *44%* |
| Magnoliales | Annonaceae | 2430 | 3 | 0.12% | 2 | 67% |
|  | Magnoliaceae | 227 | 4 | 1.76% | 0 | 0% |
| *Subtotals* | | *2657* | *7* | *0.26%* | *2* | *29%* |
| Malpighiales | Chrysobalanaceae | 530 | 1 | 0.19% | 0 | 0% |
|  | Clusiaceae | 800 | 1 | 0.13% | 0 | 0% |
|  | Euphorbiaceae | 6745 | 11 | 0.16% | 2 | 18% |
|  | Hypericaceae | 477 | 7 | 1.47% | 2 | 29% |
|  | Passifloraceae | 1035 | 3 | 0.29% | 0 | 0% |
|  | Phyllanthaceae | 2330 | 1 | 0.04% | 0 | 0% |
|  | Salicaceae | 1200 | 3 | 0.25% | 2 | 67% |
| *Subtotals* | | *13117* | *27* | *0.21%* | *6* | *22%* |
| Malvales | Bixaceae | 21 | 1 | 4.76% | 0 | 0% |
|  | Cistaceae | 270 | 7 | 2.59% | 6 | 86% |
|  | Malvaceae | 4225 | 9 | 0.21% | 3 | 33% |
|  | Thymelaeaceae | 891 | 7 | 0.79% | 1 | 17% |
| *Subtotals* | | *5407* | *24* | *0.44%* | *10* | *42%* |
| Myrtales | Combretaceae | 500 | 1 | 0.20% | 0 | 0% |
|  | Lythraceae | 650 | 1 | 0.15% | 0 | 0% |
|  | Melastomataceae | 4960 | 1 | 0.02% | 0 | 0% |
|  | Myrtaceae | 5900 | 1 | 0.02% | 1 | 100% |
|  | Onagraceae | 656 | 2 | 0.30% | 2 | 100% |
| *Subtotals* | | *12666* | *6* | *0.05%* | *3* |  |
| Nymphaeales | Nymphaeaceae | 58 | 2 | 3.45% | 2 | 100% |
| Oxalidales | Elaeocarpaceae | 635 | 1 | 0.16% | 1 | 100% |
|  | Oxalidaceae | 570 | 1 | 0.18% | 0 | 0% |
| *Subtotals* | | *1205* | *2* | *0.17%* | *1* | *50%* |
| Pinales | Pinaceae | 231 | 8 | 3.46% | 2 | 25% |
| Piperales | Aristolochiaceae | 587 | 3 | 0.51% | 0 | 0% |
|  | Piperaceae | 3615 | 1 | 0.03% | 0 | 0% |
|  | Saururaceae | 6 | 1 | 16.67% | 1 | 100% |
| *Subtotals* | | *4208* | *5* | *0.12%* | *1* | *20%* |
| Poales | Bromeliaceae | 3650 | 3 | 0.08% | 0 | 0% |
|  | Cyperaceae | 5695 | 4 | 0.07% | 2 | 50% |
|  | Eriocaulaceae | 1160 | 1 | 0.09% | 0 | 0% |
|  | Juncaceae | 442 | 2 | 0.45% | 0 | 0% |
|  | Poaceae | 11337 | 9 | 0.08% | 3 | 33% |
|  | Typhaceae | 25 | 2 | 8.00% | 2 | 100% |
| *Subtotals* | | *22309* | *21* | *0.09%* | *7* | *33%* |
| Polypodiales | Aspleniaceae | 740 | 1 | 0.14% | 0 | 0% |
|  | Dennstaedtiaceae | 240 | 1 | 0.42% | 1 | 100% |
|  | Dryopteridaceae | 1700 | 1 | 0.06% | 1 | 50% |
|  | Nephrolepidaceae | 30 | 1 | 3.33% | 0 | 0% |
|  | Onocleaceae | 5 | 1 | 20% | 0 | 0% |
|  | Polypodiaceae | 1650 | 1 | 0.06% | 0 | 0% |
|  | Pteridaceae | 1210 | 1 | 0.08% | 0 | 0% |
|  | Thelypteridaceae | 900 | 1 | 0.11% | 0 | 0% |
| *Subtotals* | | *6475* | *8* | *0.12%* | *2* | *25%* |
| Proteales | Nelumbonaceae | 1 | 1 | 100% | 1 | 100% |
|  | Platanaceae | 10 | 1 | 10% | 1 | 100% |
| *Subtotals* | | *11* | *2* | *18.18%* | *2* | *100%* |
| Ranunculales | Berberidaceae | 701 | 5 | 0.71% | 1 | 20% |
|  | Papaveraceae | 825 | 5 | 0.61% | 0 | 0% |
|  | Ranunculaceae | 2525 | 5 | 0.20% | 1 | 20% |
| *Subtotals* | | *4051* | *15* | *0.37%* | *2* | *13%* |
| Rosales | Cannabaceae | 117 | 2 | 1.71% | 1 | 50% |
|  | Moraceae | 1137 | 3 | 0.26% | 1 | 33% |
|  | Rhamnaceae | 1055 | 2 | 0.19% | 0 | 0% |
|  | Rosaceae | 2805 | 33 | 1.18% | 13 | 41% |
|  | Ulmaceae | 35 | 3 | 8.57% | 1 | 33% |
|  | Urticaceae | 2625 | 4 | 0.15% | 2 | 50% |
| *Subtotals* | | *7774* | *47* | *0.60%* | *18* | *38%* |
| Sapindales | Anacardiaceae | 873 | 6 | 0.69% | 4 | 67% |
|  | Meliaceae | 641 | 2 | 0.31% | 0 | 0% |
|  | Rutaceae | 2085 | 10 | 0.48% | 0 | 0% |
|  | Sapindaceae | 1925 | 5 | 0.26% | 4 | 80% |
|  | Simaroubaceae | 110 | 1 | 0.91% | 0 | 0% |
| *Subtotals* | | *5634* | *24* | *0.43%* | *8* | *33%* |
| Saxifragales | Altingiaceae | 13 | 1 | 7.69% | 1 | 100% |
|  | Crassulaceae | 1400 | 2 | 0.14% | 2 | 100% |
|  | Haloragaceae | 145 | 1 | 0.69% | 0 | 0% |
|  | Hamamelidaceae | 82 | 1 | 1.22% | 1 | 100% |
| *Subtotals* | | *1640* | *5* | *0.30%* | *4* | *80%* |
| Solanales | Convolvulaceae | 1880 | 4 | 0.21% | 2 | 50% |
|  | Solanaceae | 2480 | 7 | 0.28% | 2 | 29% |
| *Subtotals* | | *4360* | *11* | *0.25%* | *4* | *36%* |
| Sphagnales | Sphagnaceae | 380 | 1 | 0.26% | 0 | 0% |
| Vitales | Vitaceae | 955 | 4 | 0.42% | 3 | 75% |
| Zingiberales | Zingiberaceae | 1600 | 7 | 0.44% | 1 | 14% |
| Zygophyllales | Zygophyllaceae | 285 | 1 | 0.3% | 1 | 100% |

**References:**

1 Stevens, P. F. *Angiosperm phylogeny website. Version 14, July 2017*, <<http://www.mobot.org/MOBOT/research/APweb/>> (2001 onwards).
