# Supplementary material for: Botanical inhibitors of SARS-CoV-2 viral entry: a phylogenetic perspective
Source: Sci Rep. 2023 Jan 23;13:1244. doi: 10.1038/s41598-023-28303-x (PMC9868516; doi:10.1038/s41598-023-28303-x)

# Supplementary Material 6:

Concentration-dependent response data (2–128 μg/mL) for three select extracts (1428, 1749, and 1804) identified as hits for antiviral activity in the SARS-CoV-2 pseudovirion assay. A) viral inhibition in HEK293-ACE2 cell lines, B) percent viability (ATP assay) in HEK293-ACE2 and HaCaT cell lines, C) percent of cytotoxicity (LDH assay) in HEK293-ACE2 and HaCaT cell lines.


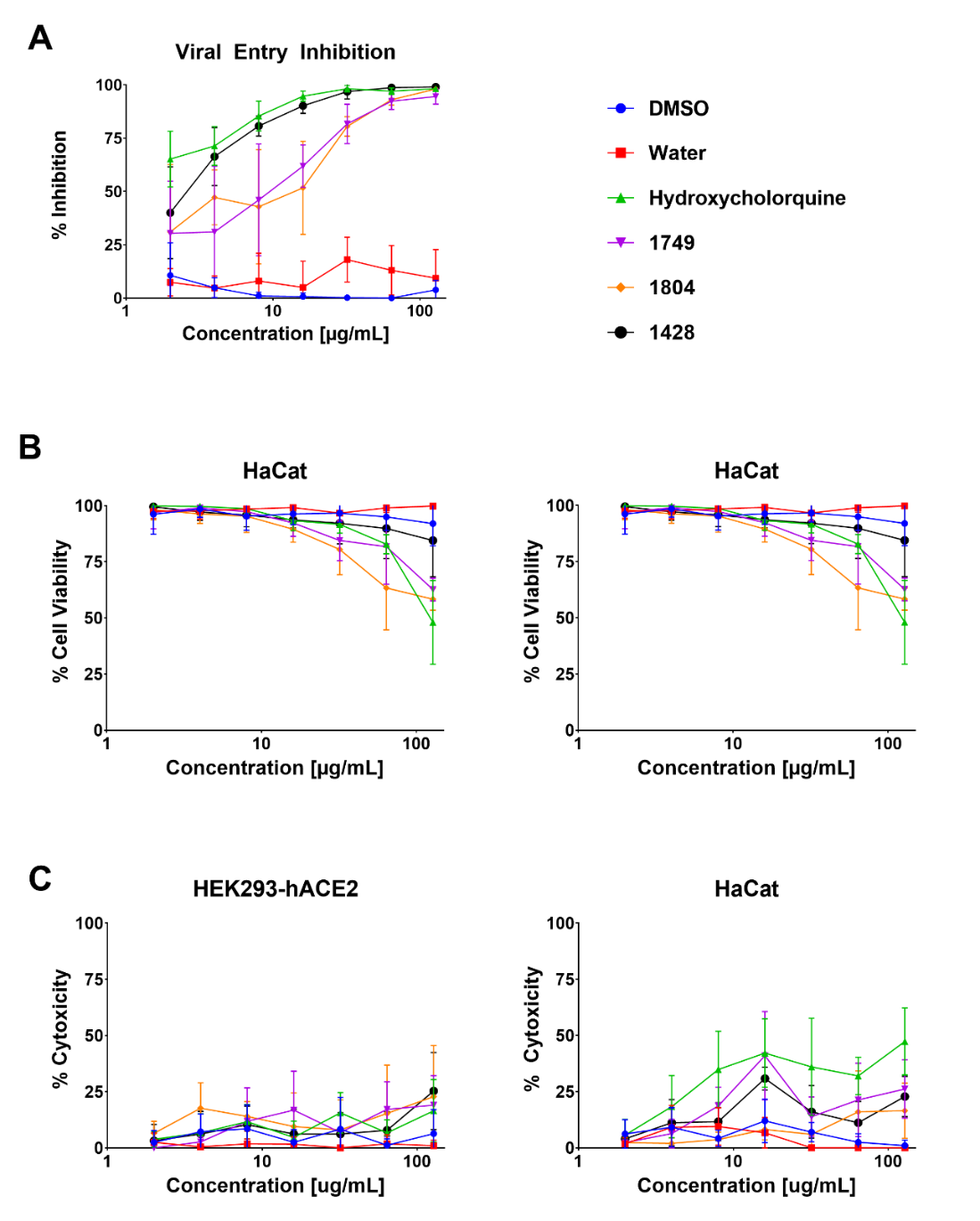

Supplement: Supplementary file 6 — Supplementary Information 6. [file 41598_2023_28303_MOESM6_ESM.docx]
