# Supplementary material for: Botanical inhibitors of SARS-CoV-2 viral entry: a phylogenetic perspective
Source: Sci Rep. 2023 Jan 23;13:1244. doi: 10.1038/s41598-023-28303-x (PMC9868516; doi:10.1038/s41598-023-28303-x)
Supplement: Supplementary file 7 — Supplementary Information 7. [file 41598_2023_28303_MOESM7_ESM.docx]

**Supplementary Material 7. Putative matches on major metabolites of 1804 and 1428 extracts.**

MSI level 1 - annotated by standard compound, MSI level 2 - annotated by MS/MS spectral matching to the GNPS spectral library, MSI level 3 - annotated by comparison of relative retention time and MS data with previous studies and characterized compound classes^1-3.^

| Detected chromatographic and spectrometric data of the tentatively identified metabolites of 1804 extracts. | | | | | | | |  |
| --- | --- | --- | --- | --- | --- | --- | --- | --- |
| peak number | identification | t_R_ (min) | calculated *m/z* [M-H]^-^ | observed *m/z* [M-H]^-^ | mass error (ppm) | molecular formula | MSI identification level ^a^ | |
| **1** | benzoyl hexose derivative | 1.94 | 329.0878 | 329.0875 | -0.93 | C_14_H_18_O_9_ | 3 | |
| **2** | caffeoyl hexose | 2.37 | 341.0878 | 341.0881 | 0.86 | C_15_H_18_O_9_ | 2 | |
| **3** | glycosidic phenolic compound | 2.44 | 371.0984 | 371.0978 | -1.53 | C_16_H_20_O_10_ | 3 | |
| **4** | A-type procyanidin dimer + prodelphinidin  (one double linkage between first and second units) | 3.27 | 879.1778 | 879.1776 | -0.23 | C_45_H_36_O_19_ | 3 | |
| **5** | A-type procyanidin trimer  (one double linkage between first and second units) | 3.66 | 863.1829 | 863.1833 | 0.48 | C_45_H_36_O_18_ | 3 | |
| **6** | caffeoylquinic acid derivatives | 4.34 | 641.1512 | 641.1514 | 0.32 | C_31_H_30_O_15_ | 3 | |
| **7** | glycosidic phenolic compound | 4.41 | [M+HCOOH-H]^-^  473.1664 | [M+HCOOH-H]^-^  473.1670 | 1.16 | C_20_H_28_O_10_ | 3 | |
| **8** | cholestane-type sterol | 6.20 | [M+HCOOH-H]^-^  509.3120 | [M+HCOOH-H]^-^  509.3145 | 4.91 | C_28_H_46_O_8_ | 3 | |
| Detected chromatographic and spectrometric data of the tentatively identified metabolites of 1428 extracts. | | | | | | | |  |
| peak number | identification | t_R_ (min) | calculated *m/z* [M-H]^-^ | observed *m/z* [M-H]^-^ | mass error (ppm) | molecular formula | MSI identification level ^a^ | |
| **1** | coumaroylquinic acid | 3.56 | 337.0929 | 337.0928 | -0.27 | C_21_H_30_O_12_ | 2 | |
| **2** | 3-*O*-feruloylquinic acid | 3.88 | 367.1035 | 367.1030 | -1.24 | C_17_H_20_O_9_ | 2 | |
| **3** | coumaroylquinic acid | 4.02 | 337.0929 | 337.0926 | -0.86 | C_16_H_18_O_8_ | 2 | |
| **4** | unidentified | 5.94 | 233.0950 | 233.0965 | 6.43 | C_11_H_19_ClO_3_ | - | |
| **5** | glycosidic triterpene derivative | 6.34 | 1643.7334 | 1643.7333 | -0.06 | C_75_H_120_O_39_ | 3 | |
| **6** | glycosidic triterpene derivative | 6.52 | 1629.7177 | 1629.7181 | 0.22 | C_74_H_118_O_39_ | 3 | |
| **7** | trihydroxy octadecenoic acid | 6.62 | 329.2333 | 329.2334 | 0.16 | C_18_H_34_O_5_ | 2 | |

**Supplemental References**

1. Sumner, L.W. *et al.* Proposed minimum reporting standards for chemical analysis Chemical Analysis Working Group (CAWG) Metabolomics Standards Initiative (MSI). *Metabolomics*. **3**, 211–221 (2007).
2. Engström M.T. *et al*. Rapid qualitative and quantitative analyses of proanthocyanidin oligomers and polymers by UPLC-MS/MS. *J Agric Food Chem.* **62**, 3390–3399 (2014)
3. Rue E.A., Rush M.D. & van Breemen R.B. Procyanidins: a comprehensive review encompassing structure elucidation via mass spectrometry. *Phytochem Rev*. **17**, 1–16 (2018)
