# Supplementary material for: Botanical inhibitors of SARS-CoV-2 viral entry: a phylogenetic perspective
Source: Sci Rep. 2023 Jan 23;13:1244. doi: 10.1038/s41598-023-28303-x (PMC9868516; doi:10.1038/s41598-023-28303-x)

# Supplementary Material 9:

Molecular Network of 1428. The annotation propagation was performed with NAP tool using GNPS, HMDB and SUPNAT databases. GNPS library matched results (green), the Consensus SMILES (blue), and the Metfrag SMILES (pink) in the *in silico* structural annotation NAP results were shown in each node.


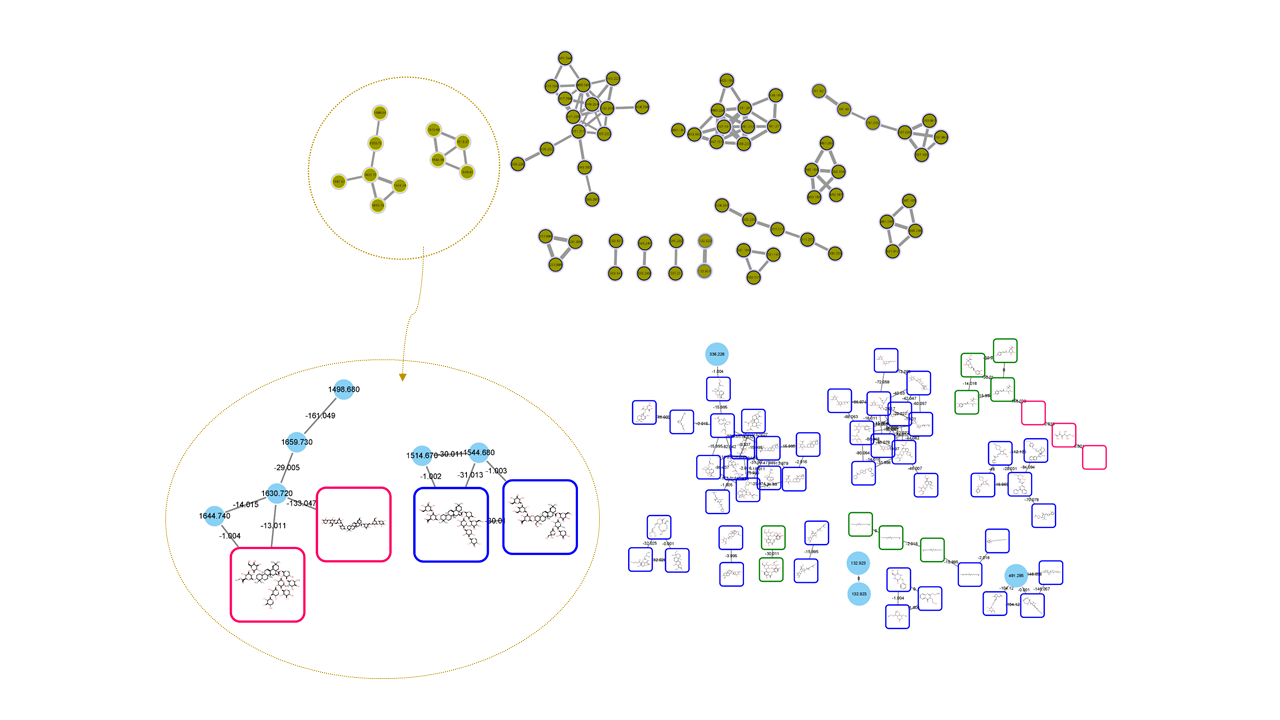

Supplement: Supplementary file 9 — Supplementary Information 9. [file 41598_2023_28303_MOESM9_ESM.docx]
